# Treatment of subclinical hyperthyroidism in patients older than 50 years: a randomized controlled study

**DOI:** 10.1530/ETJ-24-0121

**Published:** 2024-11-06

**Authors:** Bernard Goichot, François Lefebvre, Stéphane Vinzio, Anne Cailleux, Jean-Marc Kuhn, Olivier Schneegans, Bodgan Catargi, Olivier Gilly, Philippe Baltzinger, Nicolas Meyer, Philippe Caron, the investigators of the Pirahtes study

**Affiliations:** 1 Department of Endocrinology, Diabetology and Nutrition, Strasbourg University Hospital, France; 2 FMTS, Université de Strasbourg, Faculté de Médecine, France; 3 GMRC, Public Health Department, Strasbourg University Hospital, France; 4 Department of Internal Medicine, Groupe Hospitalier Mutualiste, Grenoble, France; 5 Department of Endocrinology, INSERM CIC-CRB, Rouen University Hospital, Rouen, France; 6 Department of Nuclear Medicine, ICANS, Strasbourg Cedex, France; 7 Endocrinology-Metabolic Diseases, Hôpital Saint-André, Bordeaux University, Bordeaux, France; 8 Department of Endocrinology, CHU de Nîmes, Nîmes, France; 9 iCUBE, CNRS UMR, Illkrich, France; 10 Department of Endocrinology, Metabolic diseases and Nutrition, Pôle Cardio-vasculaire et Métabolique, CHU Larrey, Toulouse; 11 Université Paul Sabatier, Inserm, Toulouse, France

**Keywords:** Atrial fibrillation, elderly, quality of life, randomized trial, subclinical hyperthyroidism, symptoms

## Abstract

**Objective:**

Subclinical hyperthyroidism (SCH) is common and associated with atrial fibrillation (AF) risk in the elderly. Current guidelines rely on a low level of evidence.

**Methods:**

Randomized clinical trial including patients 50 years and older, with thyroid-stimulating hormone (TSH) <0.4 mU/L and normal thyroid hormone concentrations. All patients showed autonomy on thyroid scan. They were randomized either to receive radioiodine (I-131) or to be monitored and treated only if they underwent AF or evolved toward overt hyperthyroidism. Primary outcome was the onset of new AF. Secondary outcomes were treatment-induced hypothyroidism rate and health-related quality of life.

**Results:**

One hundred forty-four patients (mean age: 65.3 ± 8.9 years, 76% females) were randomized, 74 to surveillance and 70 to treatment. Four patients in the surveillance group and one in the treatment group developed AF (*P* = 0.238). However, the patient who developed AF in the treatment group maintained TSH <0.4 mU/L at AF onset. A post-hoc analysis was carried out and showed that when normalization of TSH was considered, the risk of AF was significantly reduced (*P* = 0.0003). In the surveillance group, several patients showed no classical characteristics associated with AF risk, including age >65 years or TSH < 0.1mU/L. Of 94 patients treated using radioiodine, 25% developed hypothyroidism during follow-up.

**Conclusion:**

Due to recruitment difficulties, this study failed to demonstrate that SCH treatment can significantly reduce the incidence of AF in patients older than 50 years with thyroid autonomy even if all the patients who developed AF maintained TSH <0.4 mU/L. This result must be balanced with the increased risk of radioiodine-induced hypothyroidism.

## Introduction

Subclinical hyperthyroidism (SCH) emerged as a concept in the 1980s with the availability of ultra-sensitive, or second generation, thyroid-stimulating hormone (TSH) assays (1, 2, 3, 4). SCH is biochemically defined by a TSH concentration that is lower than the reference threshold while in the presence of normal free thyroid hormone (FT4 and T3 or FT3) concentrations. This situation is classically explained by the fact that the thyrotropic cells, and therefore the secretion of TSH, are particularly sensitive to small variations in the circulating concentrations of thyroid hormones and that there is a semi-logarithmic relationship between the concentration of TSH and that of free thyroid hormones, with a small variation in the latter leading to a significant variation in TSH levels. Thus, TSH levels are commonly considered to reflect the effects of thyroid hormones at the tissue level.

The importance of SCH, the prevalence of which is estimated at between 0.5% and 4% of the general population, was suggested as early as 1994 in the Sawin study (5), which showed that a decrease in TSH level was associated with an increased risk of atrial fibrillation (AF) in subjects over 60 years of age. These results were later confirmed by several other studies (6, 7, 8, 9), and though the available studies are not totally concordant, several meta-analyses suggest that in addition to AF, SCH may be associated with an increased risk of cardiovascular events and even possibly with increased mortality (10, 11, 12, 13). However, no large randomized prospective study has yet shown that treating SCH may reduce the risk of cardiovascular complications. We report here the results of a multicentric French trial designed to evaluate the effects of radio-iodine treatment on the risk of AF in patients with SCH.

## Materials and methods

PIRAHTES (**P**lace de l'**I**ode **R**adio**A**ctif dans l'**H**yper**T**hyroïdi**E S**ub-clinique/Evaluation of the interest of SCH treatment, ClinicalTrials.gov number NCT00213720) is a French multicentric clinical trial that commenced in 2006. It aims to investigate the potential cardiac benefits of radio-iodine treatment in patients with SCH.

## Trial design and patients

### Patients

Patients aged over 50 years were recruited in 20 tertiary centers in France. SCH was defined as TSH <0.4 mU/L on at least two repeated measures, using local assays at an interval between 4 and 12 weeks before randomization, with FT4 and FT3 levels in the reference range of the local assays used. Grade 1 SCH was defined as 0.1 ≤ TSH < 0.4 mU/L. Grade 2 SCH was defined as TSH <0.1 mU/L. In the case of discordance between the first and the second assays, the latter result was used to classify the patient.

Inclusion criteria: For inclusion, patients were required to have no significant cardiovascular disease apart from controlled hypertension (HT), no history of thyroid disease, and no treatment with amiodarone. Additionally, a 12-lead ECG showing a sinus rhythm was required.

Exclusion criteria included a history of AF, including paroxysmal episodes, Graves’ disease, absence of uptake on a thyroid scan (iodine saturation), history of treated hyperthyroidism, large and/or compressive goiter requiring surgery, pituitary insufficiency, concurrent severe acute illnesses, and glucocorticoids or dopaminergic agonist treatments that may interfere with TSH decrease. Patients with severe heart disease (e.g. history of arrhythmia except for isolated supraventricular or ventricular benign extrasystoles, heart failure of any etiology with a left ventricular ejection fraction <40% on an echocardiography within the 3 months preceding inclusion), and known osteoporosis, all of these conditions being considered as potentially requiring the treatment of SCH. Patients taking beta-adrenergic blockers (for hypertension mainly) were not excluded.

### Data collection

Patients were followed up every 4 months with a clinical examination, TSH and free thyroid hormone measurements, and an ECG. At each visit, they were asked to fill in three questionnaires: the HRQoL (SF-36) (14) questionnaire, anxiety and depression (HADS) (15), and a questionnaire on thyroid symptoms which was specifically designed (in French) for use in this study (manuscript in preparation). The initial protocol planned to follow the patients over 4 years. Due to difficulties with recruitment, the protocol was amended in 2014 and follow-up was extended to 6 years. Patients who were already included were asked to renew their consent.

### Hormone assays

Serum TSH, FT4, and FT3 concentrations were measured locally using commercial assays. A second confirmatory sample was required, taken at least 4 weeks and less than 3 months after the first sample. For study inclusion, patients needed to have both TSH values below 0.4 mU/L, regardless of the assay used, and normal FT4 and FT3 values according to the reference levels of the kit used.

### Thyroid scan

All patients underwent a thyroid scan using either Tc99 or I123 depending on local availability. Autonomization was defined as the presence of one (toxic adenoma) or several (multinodular goiter) nodules exhibiting high radionuclide uptake with near-null uptake in the surrounding thyroid tissue. Scans were interpreted locally by a nuclear imaging specialist. All reports were centrally reviewed.

### Randomization and procedures

Patients were randomized either to receive radioiodine treatment immediately after randomization (treatment group) or to be regularly monitored and treated only in the case of AF onset or evolution toward overt hyperthyroidism (surveillance group). Radioiodine treatment was conducted according to the usual modalities used locally. Repeated radioiodine treatment was recommended if TSH concentrations had not increased above 0.4 mU/L 8 months after the first treatment.

### End points

The primary endpoint was the onset of AF on the 12-lead ECG performed at each visit or AF diagnosed by a cardiologist during the interval between two visits. ECGs were interpreted by the local investigator. Every ECG was reviewed centrally by the same investigator, who was blind to the group of the patient, at the end of the study.

### Statistical analysis

A two-sided log-rank test with an overall sample size of 266 subjects was calculated to achieve 90% power at a 0.05 significance level, to detect a difference of 20% between a 10-year AF rate of 30% in the low TSH group vs 10% in the normal TSH group. Patients were expected to enter the study during an accrual period of 60 months with a uniform enrollment. A follow-up period of 72 months with a loss to follow-up rate of 50% in each group was assumed. This sample size was computed using NCSS-PASS 2005.

Categorical variables were described by the frequency and proportion of each category. Quantitative variables were described by the mean (± s.d.).

For the primary outcome, survival analysis was carried out. First, time-to-event curves were performed using the Kaplan–Meier method, and comparisons between groups were performed using a Cox model or the log-rank test. Secondly, a joint model taking into account the randomization group and the TSH value was performed. Then, since the joint model showed an effect of TSH on the occurrence of AF in the intention-to-treat (ITT) analysis, a post-hoc analysis was carried out: Kaplan–Meier curve and additive Lin and Ying model analysis were performed using the time-dependent variable corresponding to TSH normalization. This additive model makes it possible to estimate the effect of non-normalization of TSH on the occurrence of AF. Thirdly, linear mixed models to take into account the effect of time, assessing the effect of treatment on thyroid hormones and other outcomes were carried out.

Missing data were non-imputed.

All analyses were performed using R 4.3.1 software.

All patients were provided with standardized written information emphasizing the risk of AF and the risk of treatment-induced hypothyroidism. Prior to randomization, and after obtaining written informed consent, patients were asked to give their preference between treatment or regular clinical and biochemical follow-up. The protocol was approved by the Comité de Protection des Personnes se Prêtant à une Recherche Biomédicale according to French regulations at the time the study commenced.

## Results

### Patients

One hundred and forty-four patients were included. Patient characteristics are summarized in Table 1. The mean age was 65.3 ± 8.9 years (76% females). The mean TSH at inclusion was 0.10 ± 0.10 mU/L, 35.7% of the patients had grade 1 SCH (0.1–0.4 mU/L), and 64.3% had grade 2 SCH (TSH <0.1 mU/L). The classification between the two grades differed in 32/144 (22.2%) of the patients depending on which sample (first or second) was taken into account. All patients had a thyroid scan prior to inclusion, with Tc99 used in 132/144 (91.7%) of patients, and I123 in 9/144 (6.2%), while information was missing for three patients. The scan revealed an autonomous nodule in 21/144 (14.6%) of the patients and a multinodular goiter in 112/144 (77.8%) of the patients; it was ambiguous or unavailable in 11 patients.

**Table 1:** Characteristics of the patients at baseline. Data are presented as *n* (%) or as mean ± s.d.

Characteristics	Treatment	Surveillance	Total
*n*	70	74	144
Age (years)	65.1 ± 8.5	65.5 ± 9.2	65.3 ± 8.9
Females	51 (72.9)	59 (79.7)	110 (76.4)
First TSH (mU/L)			
1	0.10 ± 0.10	0.09 ± 0.09	0.10 ± 0.10
2	0.11 ± 0.10	0.09 ± 0.09	0.10 ± 0.10
SCH grade			
G1	27 (39.1)	24 (32.4)	51 (35.7)
G2	42 (60.90)	50 (67.60)	92 (64.3)
FT4 (pmol/L)	14.66 ± 3.13	15.86 ± 3.31	15.28 ± 3.27
FT3 (pmol/L)	5.02 ± 0.85	5.14 ± 0.79	5.09 ± 0.82
BMI (kg/m^2^)	26.10 ± 4.50	26.22 ± 4.97	26.16 ± 4.73
HR (beats/min)	73.42 ± 10.17	75.32 ± 16.36	74.42 ± 13.76
SBP (mm Hg)	130.34 ± 17.14	135.41 ± 17.21	132.97 ± 17.30
DBP (mm Hg)	76.02 ± 10.41	79.43 ± 10.93	77.78 ± 10.78
Hypertension	17 (24)	21 (28)	38 (26)
Diabetes	6 (9)	13 (18)	19 (13)
Smoking			
Current	3 (4.3)	9 (12.2)	12 (8.3)
Former	19 (27.1)	18 (24.3)	37 (25.7)
Never	48 (68.6)	47 (63.5)	95 (66.0)
HADS (0–21)			
Anxiety	7.10 ± 3.58	7.48 ± 3.74	7.30 ± 3.65
Depression	4.96 ± 2.80	5.66 ± 3.11	5.32 ± 2.97
SF36 (0–100)			
PCS	49.28 ± 7.55	47.00 ± 6.30	48.10 ± 6.99
MCS	47.24 ± 10.89	44.92 ± 11.05	46.04 ± 10.97

BMI, body mass index; DBP, diastolic blood pressure; HR, heart rate; HADS, Health Anxiety Depression Scale; MCS, mental component score; PCS, physical component score; SCH, subclinical hyperthyroidism; SBP, systolic blood pressure.

The 144 included patients were randomized, 74 to the surveillance group and 70 to the treatment group.

In the surveillance group, four patients withdrew consent after the first visit. Twenty-seven patients required treatment during follow-up, four because of the onset of AF, 21 because of evolution toward overt hyperthyroidism during follow-up, and three because they were considered to have clinical symptoms that could be attributed to SCH and were treated based on the decision of local investigators. Of the remaining 47 patients in this group who did not require treatment, 26 completed the 6-year follow-up.

In the treatment group, three patients were excluded due to their refusal of the radioiodine treatment. Sixty-seven received radioiodine treatment, of whom 38 completed the 6-year follow-up.

**Figure 1:**
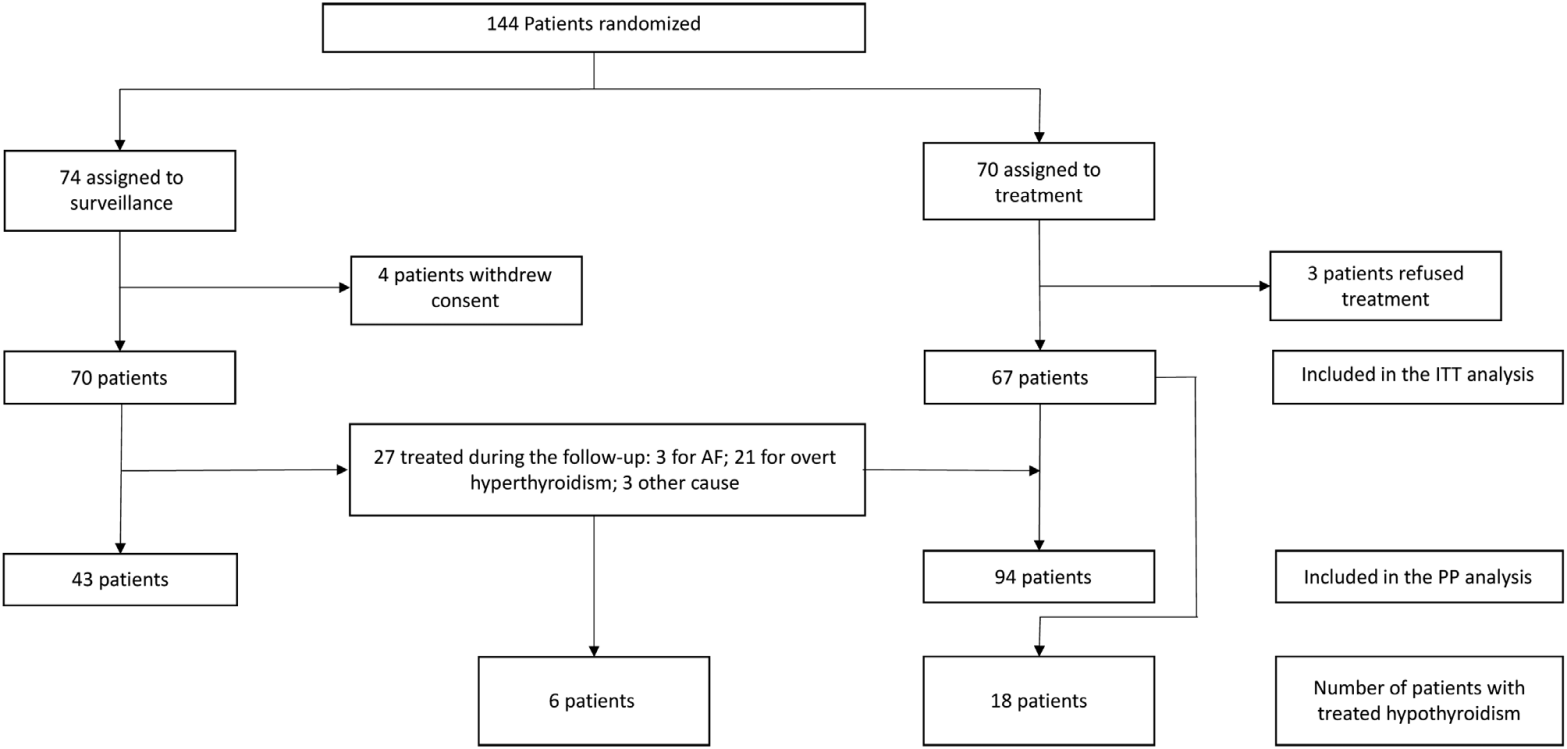
Flow-chart of the study. ITT, Intention to treat; PP, per protocol.

A flowchart (Fig. 1) shows the populations that were included in the ITT and per-protocol analysis, respectively.

### Primary outcome

**Figure 2:**
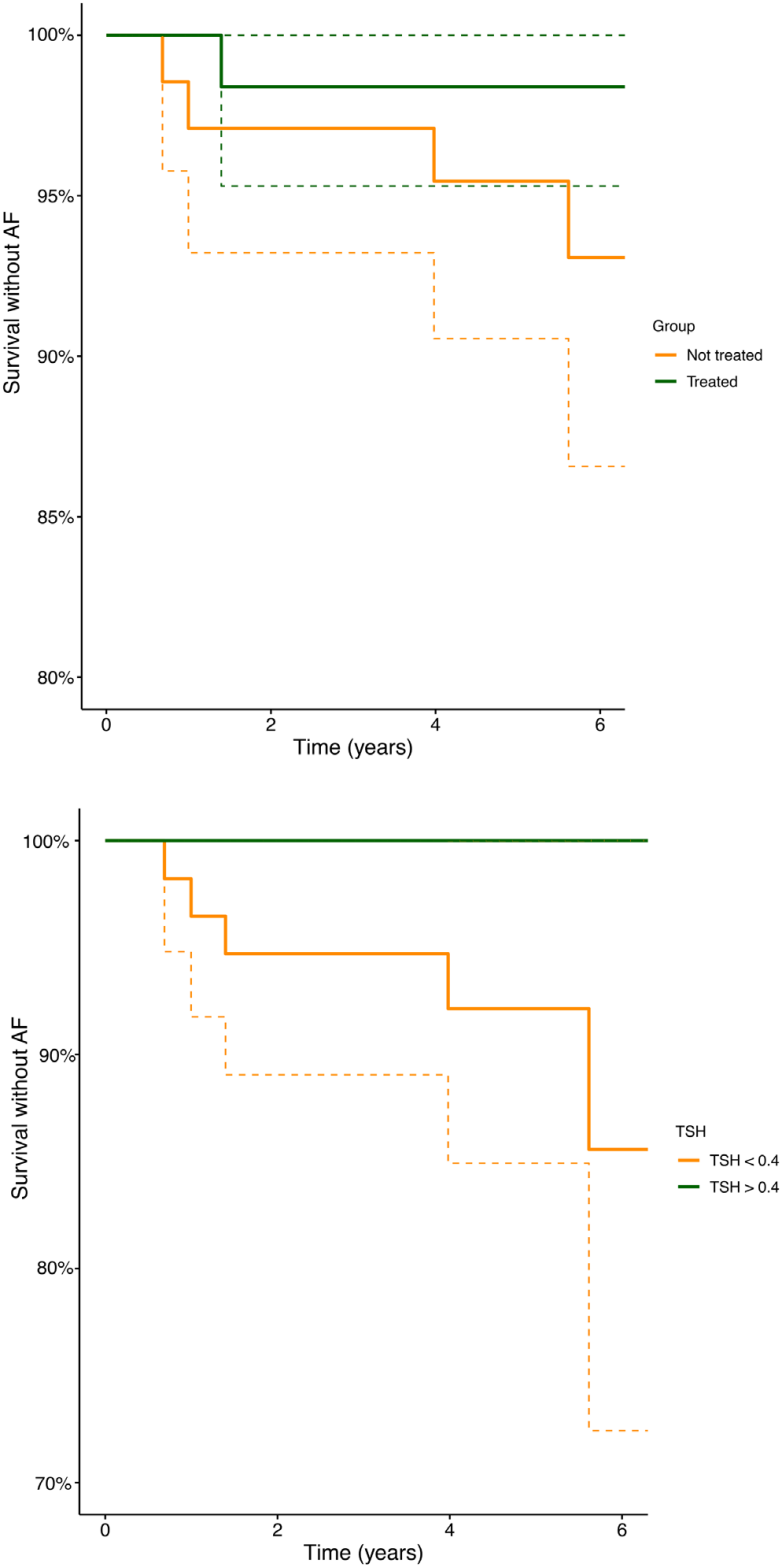
The top panel shows Kaplan–Meier survival curve for freedom of atrial fibrillation depending on the treatment or surveillance group (*P* = 0.238). The bottom panel shows Kaplan–Meier survival curve for freedom of atrial fibrillation depending on TSH concentration at the end of the study (*P* = 0.0003).

Four patients in the surveillance group developed AF during follow-up compared to only one patient in the treatment group. This difference was not significant in the ITT analysis (*P* = 0.238) (Fig. 2). Table 2 summarizes the characteristics of patients who developed AF. One of the patients was comparatively young (53 years old). About half the patients had grade 1 SCH (mildly decreased TSH) at inclusion and at the onset of AF. Several patients had associated comorbidities (HT, diabetes mellitus) that are well-recognized risk factors for AF. Importantly, the single patient who developed AF in the treatment group did not have normalized TSH at the time of AF onset. The TSH level in this patient had increased after the first radioiodine (RAI) treatment but remained below 0.4 mU/L. This patient should have been re-treated according to the protocol. When analyzing the whole population according to the objectives of treatment, which was the normalization of TSH (i.e. according to their TSH, TSH < 0.4 or TSH ≥ 0.4 in a post-hoc analysis), patients who had normalized TSH (*n* = 94) showed a statistically significant decrease in AF risk compared to those patients with persistent low TSH (*n* = 41) (Fig. 2, *P* = 0.0003). Normalization of TSH resulted in a decrease in AF risk of 2.14%/year (95% CI: 0.25–4.02), (*P* = 0.0243). The joint model failed to demonstrate a significant effect of treatment in ITT analysis regarding the occurrence of AF, but showed that an increase of 0.1 mUI/L decreased the risk of AF (HR = 0.69 (95% CI: 0.46; 0.96)).

**Table 2:** Characteristic of the patients who had AF.

	Treatment	Surveillance
*n*	1	4
Age (years)		
Mean ± s.e.m.	75	70.5 ± 8.7
Median (range)	75	73 (53–83)
TSH (mU/L)*		
1	0.02	0.61 ± 0.076
2	0.02	0.129 ± 0.074
G1/G2 (*n/n*) first TSH	0/1	1/3
G1/G2 (*n/n*) second TSH	0/1	2/2
Hypertension (*n*)	1	2
Diabetes (*n*)	1	1
Onset of AF (months)	16	4/12/36/52
TSH at AF onset (mU/L)	0.35	0.22/0.03/0.26/0.01

*values are mean ± S.E.M. G1, grade 1 (TSH: <0.1 to <0.4 mU/L); G2, grade 2 (TSH: <0.1 mU/L).

### Other endpoints

Five patients required treatment twice, and one needed a third treatment. Radioiodine activity was 404 ± 103 MBq in the treatment group and 485 ± 150 MBq in the surveillance group (NS). At the end of the study, 24 of the 94 treated patients (25.5%) had developed radioiodine-induced hypothyroidism (18 in the treatment group and six in the surveillance group).

**Figure 3:**
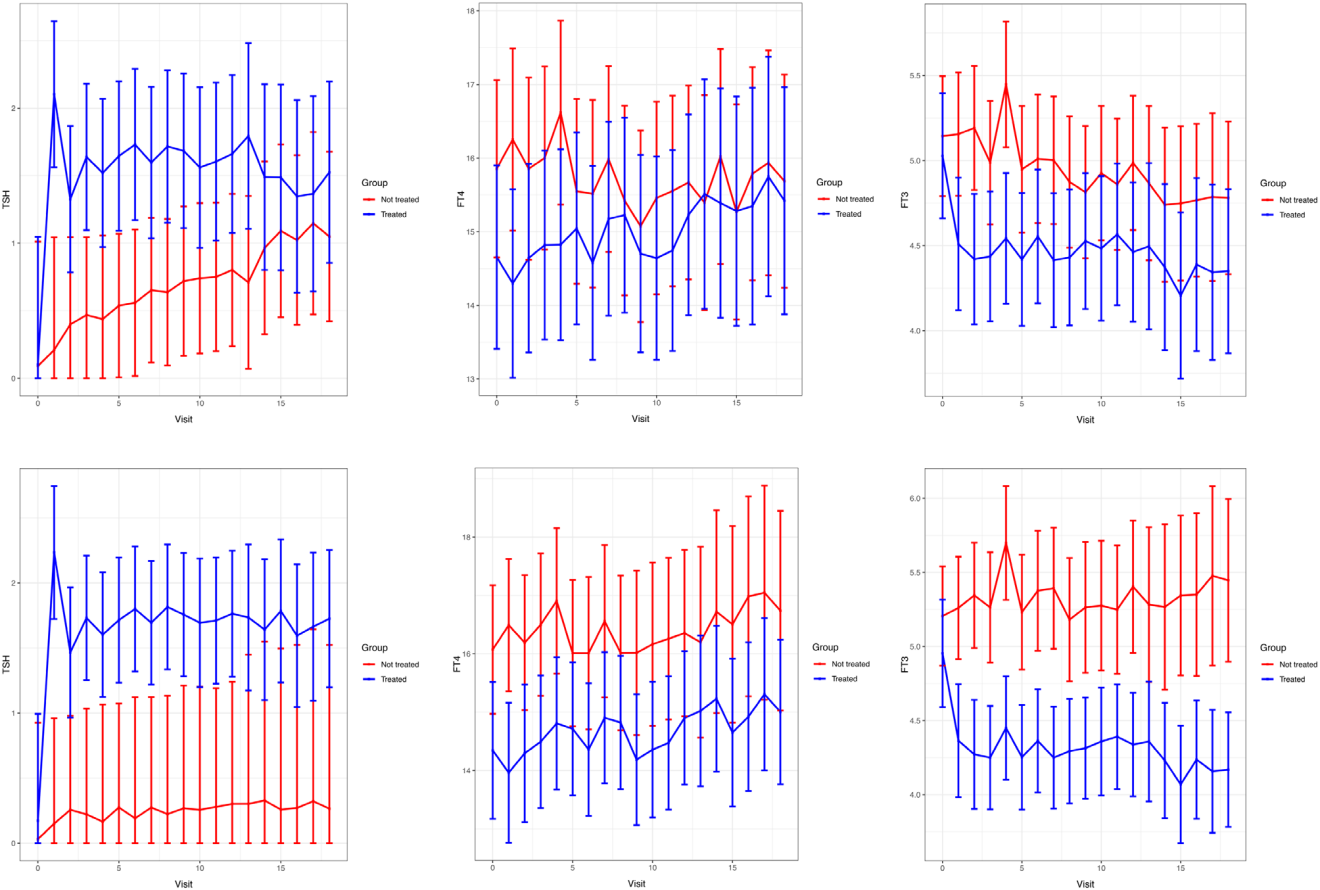
Evolution of TSH, FT4 and FT3 during the study in both groups. The error bars are 95% confidence intervals. Top panels show intention to treat analysis; bottom panels show *post-hoc* analysis.

The evolution of TSH, FT4, and FT3 concentrations in the two groups is shown in Fig. 3. In addition to the normalization of the TSH level in the treatment group, the FT3 level was significantly lower in this group compared to the surveillance group (*P* < 0.001), whereas no difference was observed for the FT4 level.

There was no difference in heart rate (HR) between the groups throughout the study, whereas weight significantly decreased in the surveillance group (−0.072 (95% CI: −0.124; −0.020) kg/visit or −1.292 (95% CI: −2.228; −0.356) kg during the study), while it remained stable in the treatment group. No differences were noted for the HAD scores between groups. There was a slight (non-significant) decrease in the physical component score (PCS) over time in both groups (meaning a worsening of this component of quality of life), whereas the mental component score (MCS) remained stable.

## Discussion

In patients over 50 years of age with subclinical hyperthyroidism and signs of autonomy on thyroid scintigraphy, radioiodine treatment that normalizes TSH reduces the risk of AF over a 6-year follow-up period. The management of subclinical hyperthyroidism is currently based on various recommendations and expert opinions, all based on a very low level of evidence (16, 17, 18, 19). The PIRAHTES study is the first RCT to demonstrate that radioiodine treatment of SCH can reduce the risk of AF. Despite several limitations discussed below, the study provides important new information on the management of SCH. First, it demonstrates that such a randomized study with a relevant clinical endpoint is feasible in the field of subclinical thyroid diseases. Despite its frequency in the general population, conducting clinical trials is difficult and several previous attempts have failed, in particular in Europe. The main reason for this failure appears to be difficulty with patient recruitment, which was also encountered in the present study. Recruitment may be difficult because these patients are, by definition, asymptomatic and are most often managed by general practitioners who are often unaware of the existence of clinical trials. More specifically, patients may be reluctant to participate due to their age and the constraints of the study protocol (consultations, etc.), but also likely to be due to difficulty in understanding the clinical question. The only well-proven complication of SCH is AF, which itself is generally a silent disease but carries potential severe risks, in particular stroke. Patients may find it difficult to assess the potential benefits of treating an asymptomatic endocrine condition (SCH) and may be reluctant to accept the principles of radio-isotopic treatment, which may be associated with specific perceived risks (radioactivity), and moreover, the irreversible nature of the treatment, including the risk of induced hypothyroidism and the subsequent need to have lifelong levothyroxine therapy. Randomized therapeutic trials in the converse endocrine situation of subclinical hypothyroidism have also encountered some of the same obstacles (20). These difficulties underline the need for research funding from government/academic funding agencies for common problems which are not of interest to the pharmaceutical industry but have potential major public health impacts.

Apart from the main results obtained, the PIRAHTES study provides additional information that could have a considerable influence on clinical practice. Most guidelines consider age (< or >65 years) and ‘grading’ based on TSH concentration (< or >0.1 mU/L) as relevant criteria for deciding whether or not to treat. Despite a limited number of events, our study suggests that the 65-year age limit is not relevant, since one of the patients who developed AF was 55 years old. Grading, at least using the threshold of 0.1 mU/L, also does not appear to be pertinent, as three of the five patients who developed AF had TSH >0.1 mU/L at the time of AF onset. Moreover, in most patients, TSH levels can fluctuate by around 0.1 mU/L, leading to them being classified as either G1 or G2 depending on the sample. This was the case for 22% of patients at inclusion and during follow-up.

One of the strengths of the study was to include only those patients with signs of thyroid autonomy on a thyroid scan. The main reason for this was to exclude TSH decrease unrelated to thyroid disease, but also to exclude patients with Graves’ disease who likely have a different natural history. At the time of protocol design, the available data, which has since been confirmed, showed that the risk of progression to overt hyperthyroidism was low in these patients compared to those with ‘autonomous’ thyroid disease (toxic adenoma or multinodular goiter) (21, 22). It would thus have been questionable to propose radical treatment with radioiodine in this context, which could potentially induce definitive hypothyroidism. The deliberate selection of patients with thyroid autonomy, who were therefore at greater risk of progressing to overt hyperthyroidism, explains why more than one third of the patients in the surveillance group required treatment, mainly because they developed overt hyperthyroidism. Thus, as suggested in recent guidelines, the cause of SCH should be taken into account in the decision to treat (19, 23).

Another important result of the present study is the rate of radioiodine-induced hypothyroidism. During the design of the study, it was not possible to reach a consensus between the clinical centers concerning therapeutic modalities. Though there was a fairly large degree of variability in the activity of I-131 administered, the rate of hypothyroidism observed was within the range of published studies (24, 25, 26). It is important to underline that the duration of observation after radioiodine treatment was limited, being 6 years in the treatment group but lower in the surveillance group, and that an unknown proportion of these patients will likely develop hypothyroidism in the coming years. Iatrogenic hypothyroidism is a crucial issue when assessing the benefit of SCH treatment. First, because the treatment of this asymptomatic condition to prevent AF carries a significant risk of creating a new chronic disease that requires definite lifelong treatment, which may be difficult to accept for patients. Secondly, because numerous studies in a real-life setting have shown that many patients taking levothyroxine do not have TSH in the normal range, with fluctuations in levels which most likely, in the case of decreased TSH, would be associated with the same risk of AF as endogenous SCH (27, 28). This could very significantly decrease the potential benefits of SCH treatment.

### Future studies

Several points remain unresolved and need to be addressed in future studies. The question of the age of patients to be treated is not simple, as the risk of AF is lower in younger subjects, in whom hyperthyroidism is often linked to Graves’ disease and not to thyroid autonomy. One of the main points that would confirm the benefit of long-term treatment in real life is to ensure that levothyroxine treatment of potential hypothyroidism induced by radioiodine treatment enables equilibrium to be achieved without overdosing. The question of radical treatment to prevent AF risk resulting in definitive hypothyroidism is also central. Future studies should therefore include an arm treated with antithyroid drugs (ideally compared to a radioiodine-treated arm which, in view of the present study, becomes the reference treatment). The target TSH values to be achieved should also be determined.

In conclusion, the PIRAHTES study demonstrates for the first time that treatment of SCH may reduce the risk of AF in patients aged more than 50 years and with thyroid autonomy. The patient age and TSH concentration (grade of SCH) do not appear to be the best criteria for the decision to treat. The benefit of treatment should also be balanced with the high rate of radioiodine-induced hypothyroidism.

## Declaration of interest

BG and PC have served as consultants and speaker for Merck Serono laboratory. BG is on the editorial board of the *European Thyroid Journal*. BG was not involved in the review or editorial process for this paper, on which he is listed as an author.

## Funding

This study was funded by the French Programme Hospitalier de Recherche Clinique (PHRC N 2004/3463).

## Authors contribution statement

BG conceived the study, raised funds, took charge of patient management, and wrote the article. FL performed the statistical analysis and reviewed and edited the article. SV contributed to study conception and design and reviewed the article. AC, JMK, OS, BC, OG, and PB took charge of patient management, collected patient data, and reviewed the article. NM contributed to the design of the study and to statistical analysis. PC contributed to the design of the study, took charge of patient management, and reviewed and edited the manuscript.
